# Parental Perceptions of Pediatric Fever From Two Medical Centers in Lebanon

**DOI:** 10.1155/ijpe/1336810

**Published:** 2025-07-24

**Authors:** Reem Eid, Ramy Touma Sawaya, Andrew Farhat, Pascale Salameh, Sarah El Yaman, Maroun Matar

**Affiliations:** ^1^Department of Pediatrics, Lebanese American University Medical Center-Rizk Hospital, Beirut, Beirut Governate, Lebanon; ^2^Gilbert & Rose-Marie Chagoury School of Medicine, Lebanese American University, Byblos, Lebanon; ^3^INSPECT-LB: Institut National de Santé Publique, Épidémiologie Clinique et Toxicologie-Liban, Beirut, Lebanon; ^4^Medical School, University of Nicosia, Nicosia, Cyprus; ^5^Faculty of Pharmacy, Lebanese University, Beirut, Lebanon

## Abstract

**Background:** Pediatric fever is considered one of the most common reasons for parents presenting to the emergency department (ED). As there is little information involving parental knowledge, management techniques, and consultation of medical professionals regarding complaints of pediatric fever in Lebanon, it is important to investigate whether the parents know how to deal with a febrile fever in child before bringing them to the ED in order to determine the need for parental educational programs for fever management and to possibly limit unnecessary hospital visits.

**Study Design:** This study recruited 191 participants to fill a questionnaire of 21 questions divided into three sections assessing parental sociodemographic characteristics, knowledge, and attitudes towards pediatric fever during presentation to the ED in two medical centers in Lebanon. The participant population was then stratified and compared on the basis of gender and knowledge level.

**Results:** When assessing participants' knowledge, 119 (62.3%) of participants had a high level of knowledge, and 72 (37.7%) had a low level of knowledge on pediatric fever. The two groups showed different approaches towards low-grade fevers with the high knowledge group presenting to the ED mostly during high-grade fevers of 39°C or more.

**Conclusion:** While the population of the current study showed similar levels of knowledge as those in studies from different parts of the world, around 50% of the participants showed higher levels of knowledge, thus proving that there is still a need for parental awareness and education regarding the causes of fever, its risks and benefits during disease progression, and appropriate ways of management.

## 1. Introduction

Fever is one of the most common chief complaints for a pediatric ED visit, as it accounts for more than 50% of the presenting cases [1]. According to the *Nelson Textbook of Pediatrics*, normal human body temperature ranges from 36.6°C to 37.9°C and any temperature above 38.0°C is considered a low-grade fever, and above 39°C a high-grade fever [2]. Although there is no general agreement, the majority of national and international guidelines define fever as being 38.0°C or higher [3–9].

Even though self-resolving viral infections are the main cause of fever in children, many parents believe that fever is dangerous and worry about the possibility of febrile seizures, brain injury, and even death as possible side effects of febrility. These ideas are supported by some medical practitioners, whose primary reason for fever reduction is often to prevent febrile convulsions [10, 11]. However, other healthcare workers would rather maintain the fever under constant control and supervision in order to effectively eliminate invading pathogens that cannot thrive at temperatures higher than 36°C–37°C [12].

It is crucial to understand that fever is not a sickness but rather a physiological reaction elevating body core temperature in response to certain pathological processes that induce the release of immunological factors and fever-inducing substances known as pyrogens that trigger the hypothalamic thermoregulatory centers [3, 13–15]. Fever also promotes the generation of neutrophils and T lymphocytes, inhibits the replication and expansion of germs and viruses, and helps in the body's acute-phase response [16, 17].

Fever is commonly interpreted by parents as a disease that needs treatment instead of a symptom or indication of illness from an underlying cause [18]. Limited parent awareness of the cause of fever and misconceptions about how fever affects their children's health can often bring significant concern and anxiety [19]. This mindset was described as “fever phobia” in 1980 by Dr. Schmitt who was the first to use the expression to represent parents' concerns regarding fever in combination with several myths about its treatment and its function in illness. Schmitt came up with a list of 10 recommendations to help people overcome their fever phobia. His main recommendation was to inform parents about the meaning of fever [20, 21] Since then, numerous studies have demonstrated the prevalence of this mindset among parents in Europe and the United States [1, 22]. An Australian review demonstrated the presence of insufficient progress in parental knowledge, beliefs, and response to febrile child, which emphasizes the necessity for organized research programs [23].

In Lebanon, only one study has assessed parental knowledge and management of fever and this was limited to one cross-sectional study of preschool children aged 5 years or less [9]. Therefore, it is important to investigate parental knowledge in managing a febrile child before bringing them to the ED in order to assess the need for parental educational programs for fever understanding, management, and medical consultation.

## 2. Methods

### 2.1. Minimal Sample Size Calculation

This prospective study employed a questionnaire to parents of febrile children presenting to the ED at Lebanese American University Medical Center (LAUMC)-Rizk Hospital (RH) and Saint John Hospital (SJH). Data was collected for approximately 2 years starting November 2022. The minimal sample size was calculated using the Epi-InfoTM software (Center for Disease Control, Atlanta, United States) and was found to be 200 participants.

### 2.2. Inclusion and Exclusion Criteria

Inclusion criteria for this study were parents with a child aged between 3 months and 16 years complaining of fever presenting to the ED at LAUMC-RH and SJH. Parents whose febrile child was younger than 3 months or older than 16 years were not included in the study. The reason for excluding infants under 3 months of age is that the American Academy of Pediatrics (AAP) recommends adequate workup to identify a cause, including blood testing and urine sampling with cultures, that will require hospital equipment and trained personnel [24]. As for the exclusion of the patients over the age of 16 years, it is per LAUMC policy that all patients over the age of 16 must be assessed by an adult physician rather than a pediatrician.

### 2.3. Data Collection

All parents of febrile children presenting to the ED at LAUMC-RH and SJH were approached directly after initial assessment by the pediatrics team. The study's aims and methods were explained in full detail, and all parents who gave written consent to participate were asked to fill in the questionnaire and return it to the pediatric team before leaving the ED, whether discharged home or admitted for further inpatient care.

The questionnaire used was written in English and translated to Arabic, the native language of Lebanon. It was distributed to parents of febrile children aged 3 months to 16 years presenting to the ED at LAUMC-RH and LAUMC-SJH. The survey consisted of 21 questions distributed over three sections. The first section utilized eight questions and focused on the sociodemographic characteristics of the participants, without gathering any identifying information such as name, medical record numbers, contact information, or specific physical characteristics. The second section contained six questions and assessed the participants' knowledge of pediatric fever. The third section used seven questions and evaluated reported parental attitudes and practices regarding fever assessment and management and the utilization of antipyretics.

### 2.4. Data Analysis

When assessing knowledge, there were six questions used to assess a participant's knowledge level, each scored with one point per correct response. The participants were then divided into two groups with those scoring between 4 and 6 as the high knowledge group and those scoring between 1 and 3 as the low knowledge group.

The data collected was analyzed using SPSS (Statistical Package for Social Sciences) software, Version 28.0. For descriptive analysis, the frequency and percentage were used since all variables were categorical variables. The association of categorical variables was assessed using the Chi-square test; in case of expected values lower than 5, the Fisher exact test was used.

The questionnaire demonstrated strong face validity, as confirmed by the biostatistics, pediatrics, and IRB teams at LAUMC and LAU.

### 2.5. Ethical Considerations

After initial assessment of the patient to provide optimal and timely medical care, the parents were approached and the study's aims and methods were explained in full detail, and they were informed of their right to decline or drop out of the study at any time. The participants were then given the questionnaire with a cover page including the full written consent form with an allocated area for achieving consent. The participants were then requested to hand in the questionnaire prior to departure from the ED, giving them enough time to read the consent form, ask any questions, or address concerns, and thoroughly fill in the questionnaire.

## 3. Results

### 3.1. Sociodemographic

In total, 191 participants completed the survey. Of these, 157 (82.2%) were females and 31 (16.2%) were males. The average age of the participants was 36.5 ± 8.81 years, while the age average for the children was 7.8 ± 4.5 years. Then, 77 (40.3%) of the participants had two children, followed by 59 (30.8%) participants who had one child, and the remaining participants had between three and five children. The largest proportion of participants (51.8%) were from Mount Lebanon, and 29.8% were from Beirut. A majority (170, 89%) of the participants had a university-level degree, 14 (7.3%) held a high school degree, and 7 (3.6%) participants did not complete high school education. Of the 191 participants presenting to the ED, 37 reported that one of the febrile child's participants was a healthcare professional. Detailed information can be seen in Table 1.

### 3.2. Knowledge

The survey included six questions to assess knowledge of each participant. Of the participants, 110 (57.6%) were able to correctly state that the most accurate site for temperature recording was the rectum. Of the participants, 103 (53.9%) knew that the temperature cutoff for fever was 38.0°C as per the AAP guidelines [25]. Then, 135 (70.7%) considered that antipyretics were to be given at a lowest temperature of 38°C as per AAP guidelines. Of the participants, 163 (85.3%) used paracetamol as their most appropriate antipyretic, while 28 (14.7%) preferred nonsteroidal anti-inflammatory drugs (NSAIDs). Of the participants, 103 (53.9%) believed that rectal antipyretics had better effects than oral counterparts of the same dose. Of the participants, 61 (31.9%) believed that oral antipyretics were more efficient than rectal counterparts of similar dose. Then, 157 (82.2%) believed that addition of another antipyretic was appropriate if the fever did not subside after the first dose. After these results were gathered and the participants' knowledge scores were tabulated, it was found that 119 (62.3%) of participants had a high level of knowledge, and 72 (37.7%) had a low level of knowledge. These results are shown in Table 2.

When asked about their biggest concern regarding fever in the pediatric population, 94 (49.2%) of participants believed that the risk of seizure is the most feared complication, 47 (24.6%) were afraid of the possibility of a more serious underlying cause, and 50 (26.2%) were afraid of the possibility of brain damage.

There was no significant difference in knowledge between mothers and fathers (*X*^2^(1, *N* = 191) = 1.78, *p* = 0.224). People from the Mount Lebanon and North Lebanon regions were more likely to have higher levels of knowledge than those in Beirut and South Lebanon (*X*^2^(4, *N* = 191) = 8.55, *p* = 0.073). Participants with more than one child were more likely to have higher knowledge levels than those with a single child (*X*^2^(1, *N* = 190) = 3.325, *p* = 0.068). However, among the population of participants with more than one child, there was no significant difference in knowledge levels (*X*^2^(4, *N* = 190) = 5.51, *p* = 0.239). If at least one of the participants was a healthcare worker, the knowledge level was found to be higher than in cases where neither parent was a healthcare worker (*X*^2^(1, *N* = 191) = 3.49, *p* = 0.044). The population found to have higher knowledge levels tended to present to the ED with high-grade fever, while participants with lower knowledge levels brought their children at both high- and low-grade fever (*X*^2^(1, *N* = 169) = 3.04, *p* = 0.058). The comparison results are shown in Table 3.

### 3.3. Reported Practices

A majority (58.6%) of the participants followed their pediatrician's recommendation when calculating the right dose of antipyretic to give their child, while 65 (34%) followed the medication leaflet instead. The remainder of participants followed pharmacist recommendations or researched the drug prior to administration. It is also important to note that none of the 191 participants followed the advice of a nonhealthcare worker such as a family member, friend, or other acquaintance. Of the participants, 161 (84.3%) would not give antipyretics after the fever subsided, while 30 (15.7%) preferred to give antipyretics even after the fever subsided. In the 91 cases when antibiotics were given at home prior to visiting the ED, 85 (93.4%) had a pediatrician's recommendation, and 6 (6.6%) followed a pharmacist's recommendation. Again, none of the antibiotics were given under the recommendation of a nonhealthcare professional. For nonmedical remedies, the most (95, 49.7%) used option was applying cold compresses on the child's head or body, followed by taking a cold water bath (39, 20.4%) or just drinking more water (24, 12.6%). Then, 16 (8.4%) of the participants opted to not use any nonmedical remedies to alleviate the fever.

Of the participants, 102 (53.4%) brought their child to the ED more than 48 h after the onset of fever, while 41 (21.5%) visited the hospital within the first 24 h, with no significant difference between the high and low knowledge level populations (*X*^2^(2, *N* = 191) = 2.20, *p* = 0.332). Of the 32 cases that required hospital admission, 18 (56.3%) presented 48 h after fever onset, while 14 (43.8%) presented 48 h before. The results are shown in Table 4.

Upon arrival to the ED, 114 (59.7%) of the presenting children had high-grade fever of 39°C or higher as measured by the medical team on site. Then, 38 (19.9%) of the presenting cases required a prescription of antibiotics. Only 32 (16.8%) of the cases needed to be admitted for inpatient care.

## 4. Discussion

This study provides a comprehensive analysis of parental knowledge and practices regarding fever management in children in Lebanon, highlighting both progress and persistent challenges. When compared to more recent literature, our findings offer a contemporary perspective on the state of Lebanese parental understanding and behavior in managing pediatric fever in Lebanon.

### 4.1. Sociodemographic Factors

Our study revealed that the majority of participants were mothers (82.2%) with an average age of 36.5 years and a high educational level, with 89% holding a university degree. This gender bias in survey participation may reflect traditional caregiving roles in Lebanese society, where mothers are more involved in child-rearing and healthcare decisions. The high prevalence of college graduates may reflect the country's emphasis on education and the potential impact of this on healthcare practices and decision-making, such as improved accessibility to evidence-based medical advice, increased health-seeking behavior, and strengthened confidence in telemedicine and home-based management.

### 4.2. Parental Knowledge and Understanding

In comparison to the literature review, where Ng et al. [26] reported that a significant percentage of participants (48.6%) were unable to accurately recognize the threshold for fever, the present study found that 53.9% of participants correctly identified the fever threshold as 38.0°C, in line with the 2024 AAP guidelines. This suggests that while awareness is relatively high, there remains room for global improvement in parental education on fever criteria. Furthermore, this result may need to be readdressed with a larger and more diverse Lebanese population in order to improve generalizability on a nationwide level. Furthermore, while the result does show promise towards an improved awareness towards pediatric illnesses, there may be a bias towards a certain range of answers as the multiple choices provided were ranged from 37.5°C to 39°C.

Regarding the most accurate site for temperature measurement, 57.6% of participants in the current study identified the rectum, which is equivalent to the 69% rate reported by Hamideh Kerdar et al. in a 2021 study conducted in Germany [27]. This indicates that despite widespread access to information, there are still notable gaps in parental knowledge, particularly regarding the accurate measurement of fever.

Additionally, the understanding that rectal antipyretics are more effective than the oral forms was held by 53.9% of participants in our study. This finding is consistent with recent research by Tantivit et al. [28], which highlighted that nearly half of the surveyed parents believed in the superior efficacy of rectal antipyretics, despite evidence showing no significant difference in effectiveness. Such misconceptions persist across different regions, underscoring the ongoing need for targeted educational interventions to change these beliefs.

The results in this study also showed that certain sociodemographic factors such as residential location and the number of children in the family may have an effect on the knowledge level of the parents. Our study showed that parents living in the North Lebanon and Metn districts had a higher level of knowledge than those in Beirut and South Lebanon. Also, parents with more than one child had higher levels of knowledge than those with a single child, which may be due to more experience dealing with children and their associated illnesses. However, further studies with larger populations are required to properly assess these results and what factors may lead to such conclusions.

Furthermore, the result that participants with higher levels of knowledge were more likely than those with low knowledge levels to delay ED visits when the child had low-grade fever, but still present early with high-grade fever may show that fever phobia was more prominent in both populations with increasing severity of the fever, while participants that were more familiar with the side effects of fever were less concerned with low-grade fever.

### 4.3. Practices and Decision-Making

Parental practices in fever management continue to reflect both adherence to medical guidelines and anxiety-driven behaviors. In our study, 49.2% of participants identified the risk of febrile seizures as their main concern, aligning with findings from a 2001 study by Crocetti M et al. [29], in which 32% of participants cited seizures as the most significant harm associated with fever. This concern remains a significant driver for ED visits, despite the low incidence and generally benign nature of febrile seizures, as highlighted in contemporary pediatric literature [30–33].

The preference for paracetamol as the antipyretic of choice, observed in 85.3% of our participants, mirrors the findings of a 2005 study conducted by Betz MG et al. [1], where 98% of participants reported using paracetamol for fever management. This widespread use suggests that parents are largely adhering to recommendations for antipyretic use. Furthermore, the ease of use and acquisition, cheaper cost, and widespread availability of paracetamol in its different dosage forms may also promote its appropriate use amongst people of different sociodemographic backgrounds.

Interestingly, 15.7% of participants in our study continued to administer antipyretics even after the fever had subsided. This behavior is indicative of lingering “fever phobia,” a phenomenon well-documented in studies like the 2006 review by Walsh and Edwards [23] that highlights the persistent overtreatment of fever due to parental anxiety, emphasizing that despite advances in medical knowledge, fever phobia continues to influence parental behaviors. Moreover, the finding that 82.2% of participants believed in administering a second antipyretic if fever did not subside reflects the adherence to current guidelines by the AAP, which states that alternating antipyretic use when fever does not subside is beneficial if antipyretics are administered at proper doses and time intervals [3].

The study found that more than half of the participants (53.4%) delayed presenting to the ED for more than 48 h after the onset of fever, which contrasts with findings from previous studies where participants often sought immediate care [34, 35]. This could suggest a shift towards more cautious monitoring before seeking emergency care, possibly due to increased availability of telemedicine or advice from pediatricians, as 66% of participants contacted their pediatrician before visiting the ED, a proactive behavior suggesting a growing reliance on telemedicine as a first step in managing pediatric fever, which may help reduce unnecessary ED visits, as mentioned by Franklin et al. [36].

While 59.7% of children presented with high-grade fever (≥ 39°C), the lack of significant differences in ED visit timing between participants with high and low knowledge levels in our study suggests that factors beyond mere knowledge, such as parental anxiety or past experiences, play a critical role in decision-making.

## 5. Conclusions

The findings from this study highlight the need for continuous parental education on fever management. While some progress has been made in aligning parental knowledge with medical guidelines [9], as evident by the increased percentage of parents correctly identifying the fever threshold and appropriately initiating treatment for fever at home prior to visiting the ED, misconceptions and anxiety-driven practices remain prevalent. Healthcare providers should prioritize clear, consistent messaging about the natural course of fever, the appropriate use of antipyretics, and the limited risks associated with fever.

Public health campaigns should focus on demystifying fever, emphasizing that it is a symptom rather than a disease, and providing participants with evidence-based practices to reduce unnecessary ED visits. By addressing these gaps, we can improve the overall management of fever in children and reduce the burden on emergency healthcare services.

In conclusion, while many participants in this study demonstrated a reasonable level of knowledge about fever, significant gaps remain, particularly regarding the use of antipyretics and the perceived dangers of fever. Addressing these gaps through targeted education and improved communication is crucial for promoting better fever management practices and reducing unnecessary ED visits.

## Figures and Tables

**Table 1:** Sociodemographic characteristics of participants.

**Participants**	**N** ** (%)**
Kinship to patient:	
Father	31 (16.2)
Mother	157 (82.2)
Age class (years):	
≤ 25	2 (1.2)
26–30	20 (12.1)
31–35	63 (37.9)
36–40	47 (28.3)
> 40	34 (20.5)
Average age of participants (years):	36.5 ± 8.81
Number of children:	
1	59 (30.9)
2	78 (40.8)
3	42 (22.0)
4	11 (5.8)
5	1 (0.5)
Children's average age (years):	7.8 ± 4.5
Residential area:	
Beirut	57 (29.8)
Mount Lebanon	99 (51.8)
South Lebanon	15 (7.9)
North Lebanon	13 (6.8)
Bekaa	7 (3.7)
Educational level	
Elementary school	2 (1.0)
Intermediate school	5 (2.6)
High school	14 (7.3)
University	170 (89.0)
Are any of the participants a medical professional?	
Yes	37 (19.4)
No	154 (80.6)

**Table 2:** Knowledge-based questions and parental knowledge level.

**Participants**	**N** ** (%)**
Most suitable site for temperature recording:	
The armpit	12 (6.3)
The rectum	110 (57.6)
Groin crease	1 (0.5)
The mouth	9 (4.7)
The ear	52 (27.2)
On the forehead	7 (3.7)
Fever cutoff temperature:	
37.5°C	21 (11.0)
38.0°C	103 (53.9)
38.3°C	17 (8.9)
38.5°C	39 (20.4)
39°C	11 (5.8)
Temperature for antipyretics administration:	
< 38°C	13 (6.8)
38–38.5°C	135 (70.7)
38.6–38.9°C	27 (14.1)
39°C and above.	16 (8.4)
Most appropriate antibiotic:	
Paracetamol (Panadol, Adol, and Tylenol)	163 (85.3)
NSAID (Brufen, Advil, and Profinal)	28 (14.7)
Aspirin	0 (0)
Antibiotic	0 (0)
Are rectal drugs more efficient than oral drugs?	
Yes	103 (53.9)
No	61 (31.9)
Not sure	27 (14.1)
Can we administer another antipyretic if a fever does not subside?	
Yes	157 (82.2)
No	34 (17.8)
Knowledge level:	
Low	71 (37.8)
High	117 (62.2)
Biggest concern from pediatric fever:	
Risk of seizure	94 (49.2)
Possibility of serious illness	47 (24.6)
Brain damage	50 (26.2)

**Table 3:** Comparison of sociodemographic populations on the basis of knowledge level.

**Sociodemographic factor**	**Low knowledge**	**High knowledge**	**n**	**X** ^2^	**p** ** value**
Parental kinship					
Father	15	16	31	1.782	0.182
Mother	56	101	157		
Number of children					
1	28	31	59	5.507	0.239
2	24	53	77		
3	15	27	42		
4	4	7	11		
5	1	0	1		
Residential area					
Beirut	28	29	57	8.55	0.07
Mount Lebanon	28	71	99		
South Lebanon	8	7	15		
North Lebanon	5	8	13		
Bekaa	3	4	7		
Are any of the participants a healthcare worker?					
Yes	9	63	72	3.49	0.44
No	28	91	119		
Temperature grade of child					
High grade (≥ 39.0°C)	40	74	114	3.04	0.058
Low grade (38.0–38.9°C)	27	28	55		

**Table 4:** Participant practices on fever.

**Participants**	**N** ** (%)**
Nonmedical remedy preference	
Cold compresses	95 (49.7)
Ice pack	17 (8.9)
Cold bath	39 (20.4)
Drinking more water	24 (12.6)
None	16 (8.4)
Choosing antipyretic dose according to:	
Pediatrician's order	112 (58.6)
Pharmacist's recommendation	9 (4.7)
The medication leaflet	65 (34.0)
An acquaintance's recommendation	0 (0)
Research of the recommended dose	5 (2.6)
Administration of antipyretics even after fever subsided:	
Yes	30 (15.7)
No	161 (84.3)
Presentation to ED after fever onset:	
First 24 h	41 (21.5)
Between 24 and 48 h	48 (25.1)
After 48 h	102 (53.4)
Pediatrician contact prior to visit to ED:	
Using telemedicine (texting or phone call)	126 (66.0)
In-person at the clinic	32 (16.8)
No contact with pediatrician	33 (17.3)
Antibiotic prescription:	
From pediatrician	85 (44.5)
From pharmacist	6 (3.1)
From acquaintance	0 (0)
No antibiotics given	100 (52.4)

## Data Availability

The data that support the findings of this study are available from the corresponding author upon reasonable request.
